# Supplementary material for: CAR-T cell-mediated depletion of immunosuppressive tumor-associated macrophages promotes endogenous antitumor immunity and augments adoptive immunotherapy
Source: Nat Commun. 2021 Feb 9;12:877. doi: 10.1038/s41467-021-20893-2 (PMC7873057; doi:10.1038/s41467-021-20893-2)
Supplement: Supplementary file 1 — Supplementary Information [file 41467_2021_20893_MOESM1_ESM.pdf]

Supplementary Information

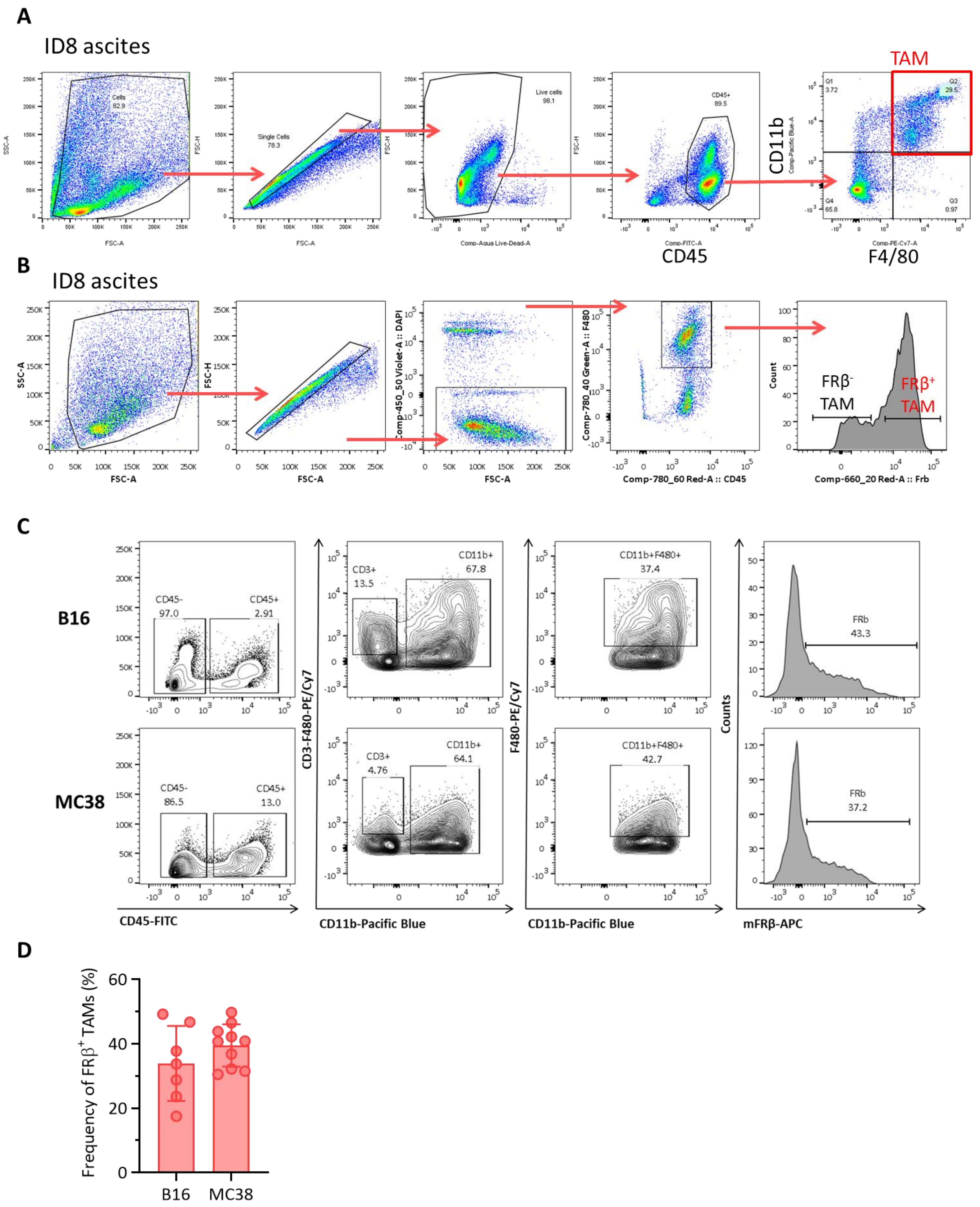

Supplementary Figure 1. Gating strategy for FACS analysis and sorting of TAMs and FRβ expression in TAMs from different tumor origins (**A**) Gating strategy for peritoneal TAMs from ID8 ovarian tumor-bearing C57BL/6 mice (defined as live/$CD45^+CD11b^+F4/80^+$). (**B**) Gating strategy to sort $FR\beta^-$ and $FR\beta^+$ TAMs from ID8 ovarian tumor-bearing C57BL/6 mice used for the assays presented in Fig. 2a, b, c, g, h; 3b, c; Supplementary Fig. 4a-f; and Supplementary Fig. 5a-f. (**C**) C57BL/6 mice were implanted subcutaneously (s.c.) with $0.5x10^6$ B16 melanoma or MC38 colon adenocarcinoma cells. 14 days later, tumors were collected, mechanically dissociated and analyzed by FACS. Representative gating strategy and FRβ expression in TAMs (defined as live/$CD45^+CD11b^+F4/80^+$) from B16 (upper panel) or MC38 (lower panel) (**D**) Frequency of $FR\beta^+$ TAMs is represented as mean ± SD (B16 n=7 tumors, MC38 n=10 tumors). Source data are provided as a Source Data file.

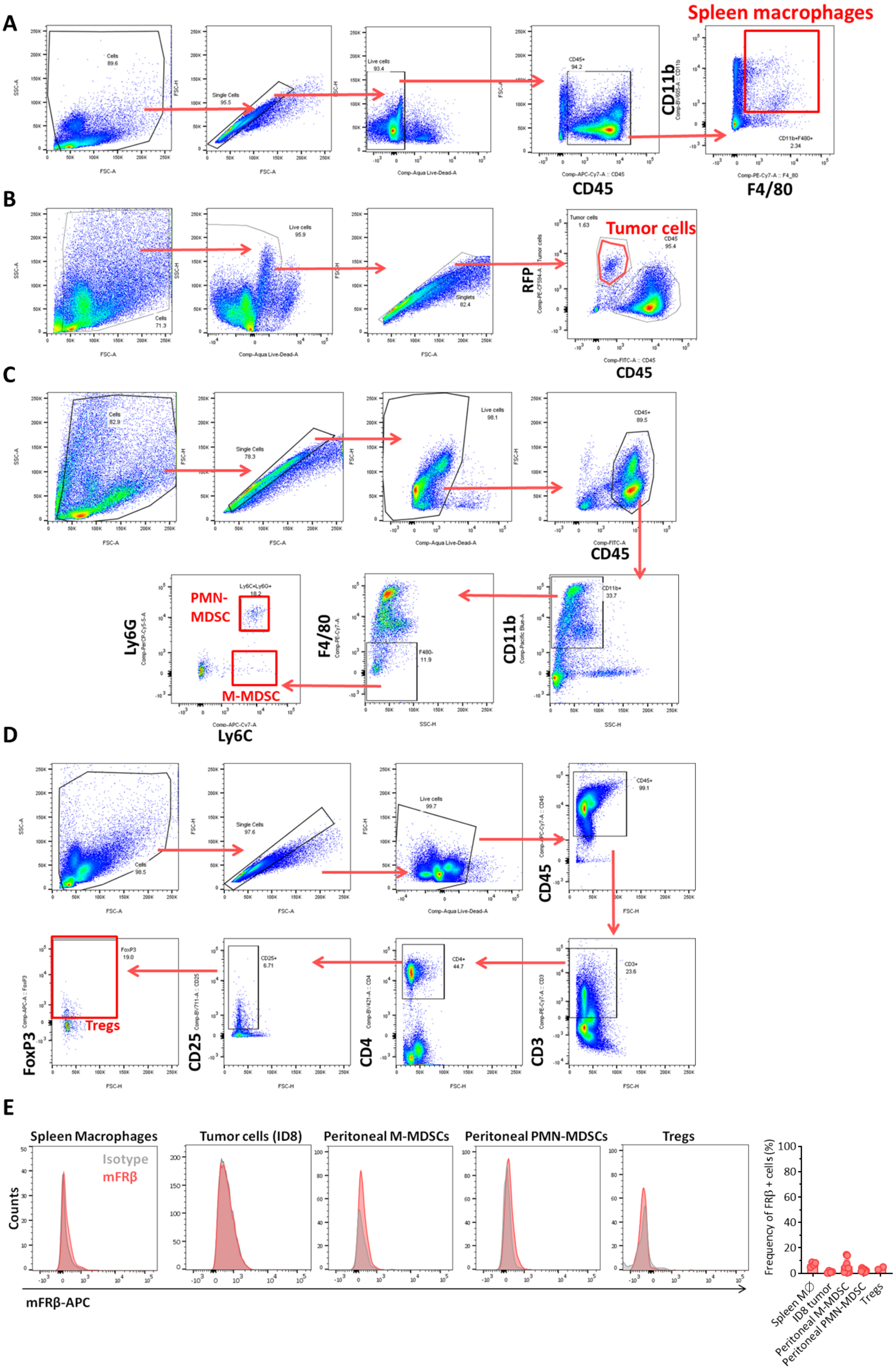
A
Spleen macrophages
CD11b
CD45
F4/80
B
Tumor cells
RFP
CD45
C
CD45
PMN-MDSC
M-MDSC
Ly6G
F4/80
CD11b
Ly6C
D
CD45
Tregs
FoxP3
CD25
CD4
CD3
E
Spleen Macrophages
Tumor cells (ID8)
Peritoneal M-MDSCs
Peritoneal PMN-MDSCs
Tregs
Isotype
mFRβ
Counts
mFRβ-APC
Frequency of FRβ + cells (%)
Spleen MØ
ID8 tumor
Peritoneal M-MDSC
Peritoneal PMN-MDSC
Tregs

Supplementary Figure 2. Gating strategy for FACS analysis and FRβ expression in the TME. Representative gating strategies shown for (**A**) spleen macrophages (defined as live/$CD45^+CD11b^+F4/80^+$), (**B**) peritoneal ID8 tumor cells (defined as live/$CD45^-RFP^+$) (**C**) peritoneal monocytic myeloid-derived suppressor cells (M-MDSCs) (defined as live/$CD45^+CD11b^+F4/80^-Ly6C^+Ly6G^-$) and polymoprhonuclear (PMN)-MDSCs (defined as live/$CD45^+CD11b^+F4/80^-Ly6C^+Ly6G^+$), and (**D**) regulatory T cells (Tregs). (**E**) FRβ expression in macrophages obtained from spleen, ID8 tumor cells, peritoneal MDSCs, and Tregs. Representative histograms (grey-isotype, red-anti-mFRβ, left panel) and frequency of $FR\beta^+$ cells (right panel) ± SD (Spleen and ID8 n=4 mice, M-MDSCs n=10 mice, PMN-MDSCs n= 8 mice, Tregs n=3 mice) are shown. Source data are provided as a Source Data file.

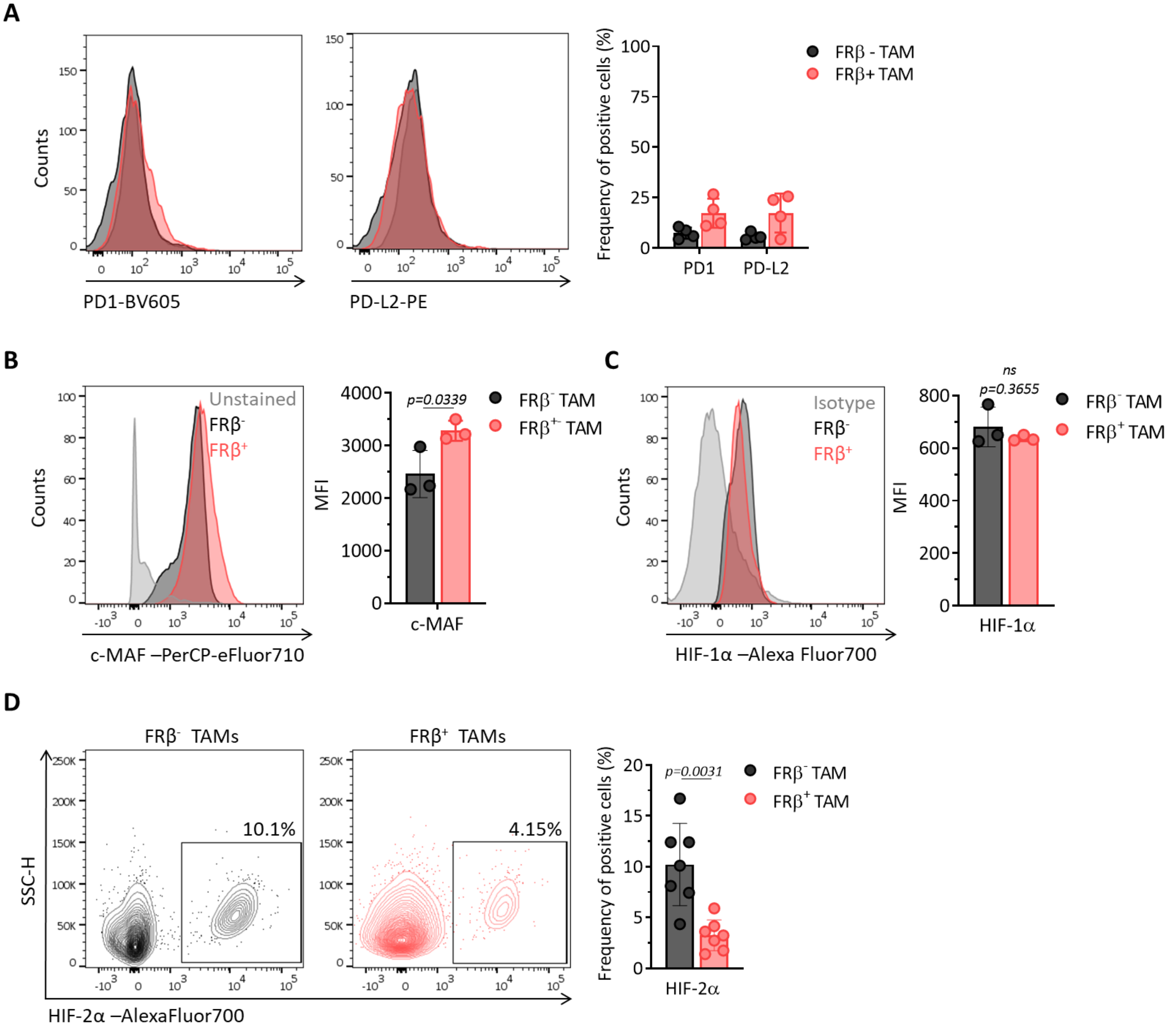

Supplementary Figure 3. Phenotypic characterization of FRβ⁺ TAMs. (**A**) Expression of checkpoint immune molecules in TAMs. Expression of PD1 and PD-L2 in FRβ⁻ and FRβ⁺ TAMs (gated on live, CD45⁺CD11b⁺F4/80⁺). Representative histograms (grey-isotype, black-FRβ⁻ TAMs, red-FRβ⁺ TAMs, left and middle panel) are shown. Frequencies of positive cells are represented as mean ± SD (n=4 mice, right panel). (**B**) Expression of c-MAF in FRβ⁻ and FRβ⁺ TAMs as detected by flow cytometry. Representative histograms (grey-unstained, black-FRβ⁻ TAMs, red-FRβ⁺ TAMs, left panel) and MFI (right panel) ± SD (n=3 mice) are shown. (**C**) Expression of HIF-1α in FRβ⁻ and FRβ⁺ TAMs as detected by flow cytometry. Representative histograms (grey-isotype, black-FRβ⁻ TAMs, red-FRβ⁺ TAMs, left panel) and MFI (right panel) ± SD (n=3 mice) are shown. (**D**) Expression of HIF-2α in FRβ⁻ and FRβ⁺ TAMs. Representative flow plots are shown (left and middle panel) and frequencies of positive cells are represented as mean ± SD (n=7 mice, right panel). *p-values* by a paired two-tailed t-test are indicated. Source data are provided as a Source Data file.

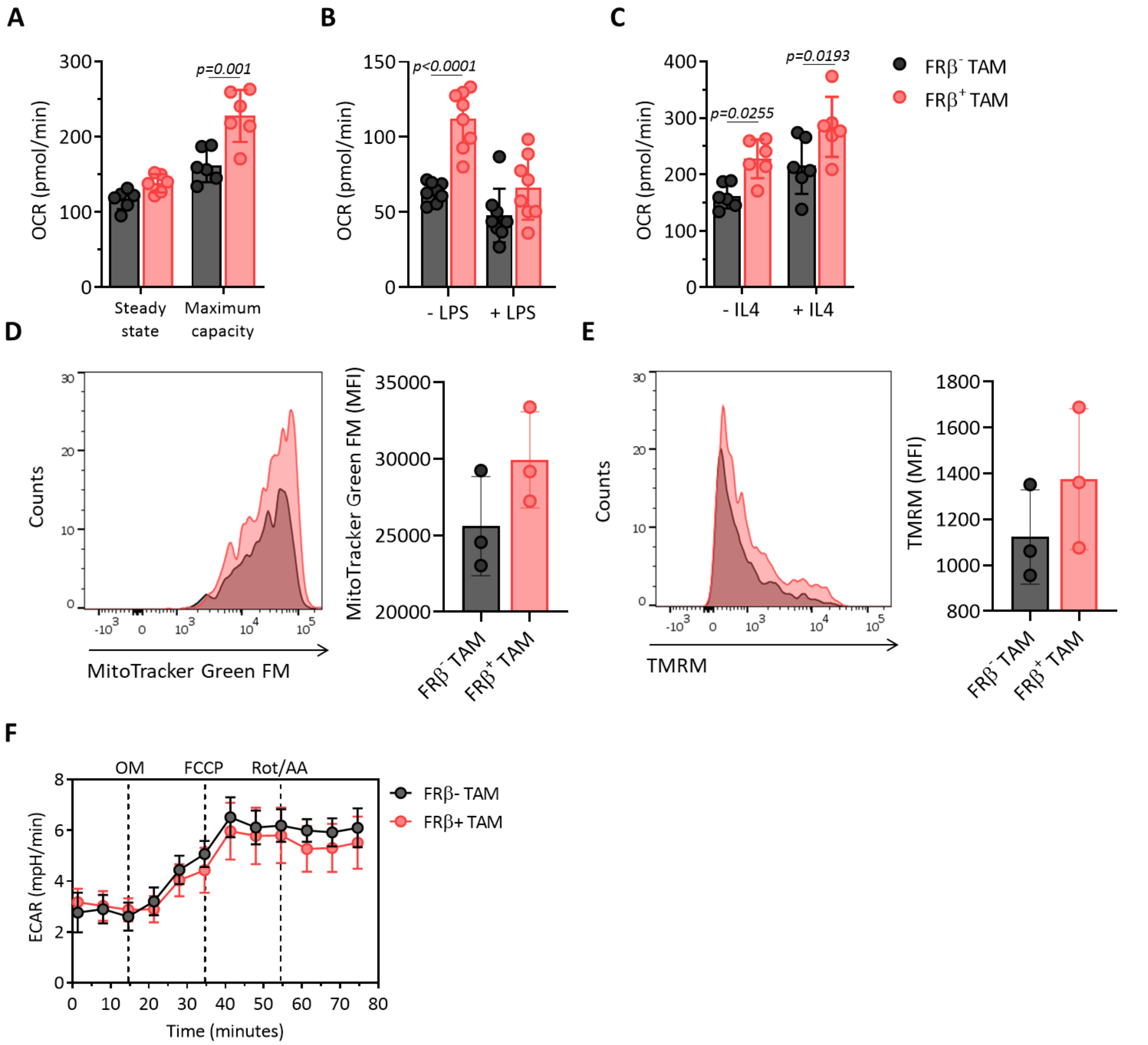

Supplementary Figure 4. Metabolic differences between FRβ^{-} and FRβ^{+} TAMs. (**A**) Oxygen Consumption Rates (OCR) of flow-sorted FRβ^{-} and FRβ^{+} TAMs after one day in culture in response to mitochondrial inhibitors added at indicated times as detected by a Seahorse assay. Basal OCR and maximum respiratory levels are represented as mean ± SD (n=6 replicates). (**B**) Maximum Oxygen Consumption Rates (OCR) of flow-sorted FRβ^{-} and FRβ^{+} TAMs after one day in culture in the presence or absence of 5ng/mL LPS (n=8) or (**C**) 20ng/mL mIL-4. Mean ± SD (n=6 replicates) is represented. Representative of two independent experiments. Intensity of (**D**) MitoTracker Green FM fluorescence and (**E**) tetramethylrhodamine, methyl ester (TMRM) as determined by flow cytometry in FRβ^{-} and FRβ^{+} TAMs (gated on live, CD45^{+}CD11b^{+}F4/80^{+}). Representative histograms (black-FRβ^{-} TAMs, red-FRβ^{+} TAMs, left panel) and MFI are represented as mean ± SD (n=3 replicates, right panel). (**F**) Extracellular acidification rates (ECAR) of flow-sorted FRβ^{-} and FRβ^{+} TAMs after one day in culture in response to mitochondrial

inhibitors added at indicated times as detected by a Seahorse assay represented as mean ± SD (n=6 replicates). *p-values* by a two-way ANOVA with Sidak's multiple comparison test are indicated. Source data are provided as a Source Data file.

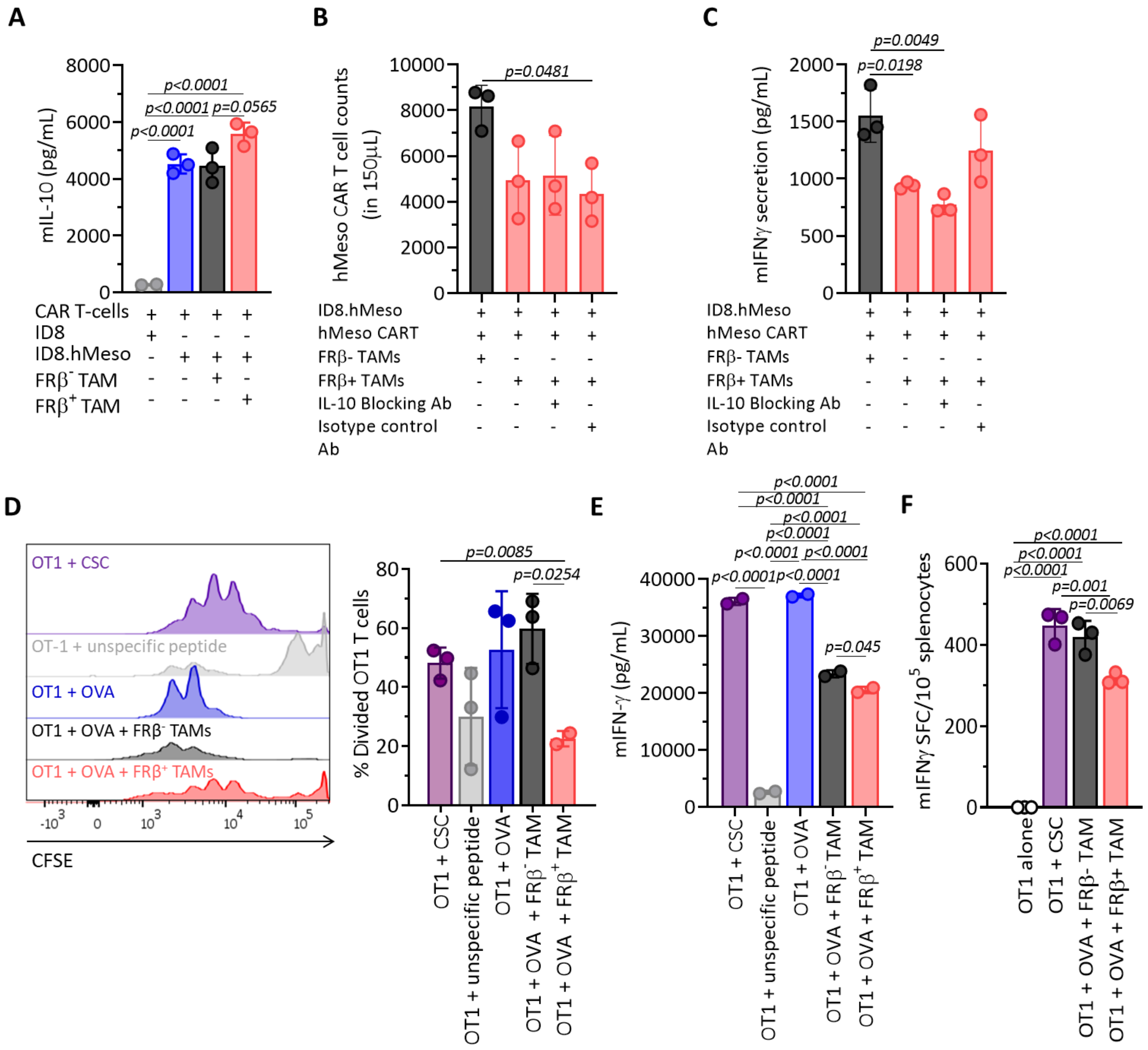

Supplementary Figure 5. FRβ^{+} TAMs suppress antigen-specific T cell responses. (**A**) hMeso-specific mouse CAR-T cells were co-cultured with ID8 or ID8.hMeso target cells in the presence of flow-sorted FRβ^{-} or FRβ^{+} TAMs for 72 hours. mIL-10 levels in 72 hour supernatants as detected by ELISA. Mean ± SD of triplicates is shown. (**B**-**D**) hMeso-specific mouse CAR-T cells were co-cultured with ID8.hMeso target cells and flow-sorted FRβ^{-} or FRβ^{+} TAMs in the presence of mIL-10R blocking antibody or the appropriate isotype control for 72 hours. (**B**) hMeso CAR-T cell counts at the end of the experiment are represented ± SD of triplicates. (**C**) mIFN-γ in 72 hour supernatants as detected by ELISA. Mean ± SD of triplicates is shown. (**D**-**F**) OVA-specific OT-1 bulk splenocytes labeled with CFSE (a fluorescent dye that is diluted with cell proliferation) were cultured in the presence of specific OVA$_{257\text{-}264}$ or unspecific peptides at 1µM for 72 hours. (**D**) Histograms showing CFSE staining on T cells (gated on live/CD3^{+} cells, left panel) as well as mean percentages of divided cells ± SD, representative from two independent experiments (right panel, n=3 replicates) are shown. (**E**) mIFN-γ secretion by OT-

1 T cells as detected by ELISA. Mean ± SD of duplicates is represented. (**F**) Number of mIFN-γ-producing cells after overnight co-culture as assessed by ELISPOT assay. Mean ± SD is represented (n=3 replicates). Representative data of two independent experiments. (**A**-**F**) *p-values* by a one-way ANOVA with Tukey's multiple comparison test are indicated. CSC, cell stimulation cocktail. Source data are provided as a Source Data file.

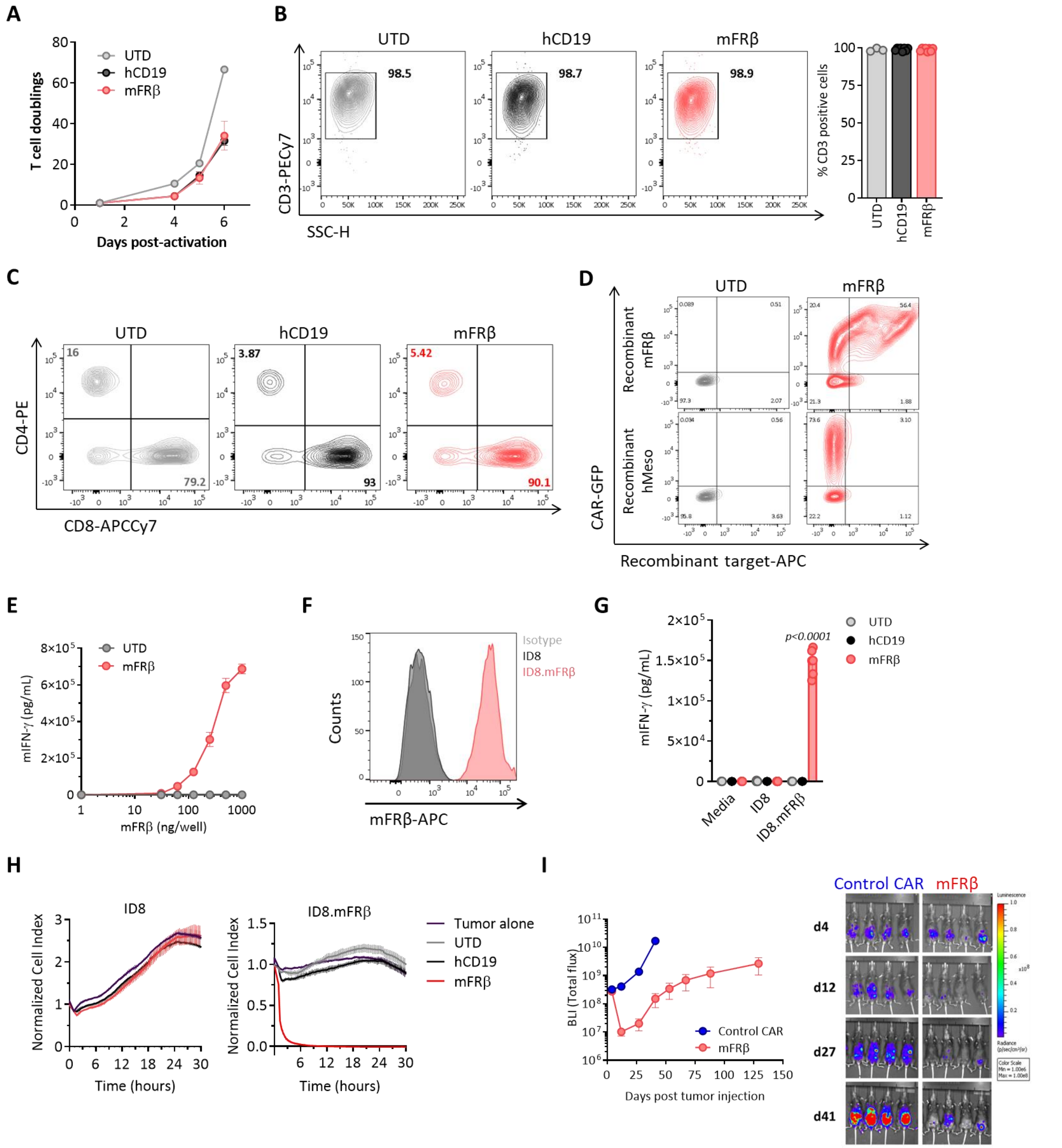

Supplementary Figure 6. Mouse FRβ CAR-T cells show antigen-specific reactivity in an engineered CAR-targetable tumor model. (**A**) T cell expansion of untransduced (UTD), hCD19 and mFRβ mouse CAR-T cells. Mean ± SD (n=3 independent expansions) is shown. (**B**) CD3 expression (Y-axis) on expanded UTD or hCD19 and mFRβ mouse CAR-T cells. Representative flow plots (left panel) and mean ± SD (right panel, UTD n=3, hCD19 and mFRβ n=8) are shown. Each dot represents an independent expansion. (**C**) CD8 (X-axis) versus CD4 (Y-axis) expression on $CD3^+$ T cells at day 5 of expansion. Representative flow plots are shown.

(**D**) mFRβ mouse CAR-T cells or UTD T cells were incubated in the presence of biotinylated recombinant mFRβ or biotinylated recombinant hMeso as control and stained with SA-APC to detect antigen binding specificity (Y-axis). Expression of GFP (X-axis) was used as a transduction marker. Representative flow plots are shown. Levels of secreted mIFN-γ as detected by ELISA in the supernatants of mFRβ CAR-T cells stimulated with (**E**) increasing concentrations of immobilized recombinant mFRβ (n=3 replicates), or (**G**) following overnight co-culture of mouse CAR-T cells and parental or mFRβ-enginereed ID8 target cells (n=6 replicates). Concentration means ± SD are represented. *p-values* by a two-way ANOVA with Tukey's multiple comparison test are indicated. (**F**) Expression of mFRβ in ID8.mFRβ cells as detected by flow cytometry. Representative histogram is shown (grey-isotype, black-ID8, red-ID8.mFRβ). (**H**) Real time cytotoxicity assays in ID8 (left panel) and ID8.mFRβ (right panel) target cells performed using xCelligence technology. Co-cultures were established at a 1:1 E:T ratio. Mean ± SD of triplicates is represented. Representative of at least five independent experiments. (**I**) C57BL/6 mice were implanted i.p. with $5x10^6$ ID8.mFRβ-RFPfLuc tumor cells and treated with $5x10^6$ mFRβ or control CAR^+ T cells on days 4, 10, 17, 31, and 38 following tumor injection. Tumor progression was monitored by bioluminescent imaging. Error bars represent mean ± SD (left panel, n=8 per group). Images from representative mice are also shown (right panel). Source data are provided as a Source Data file.

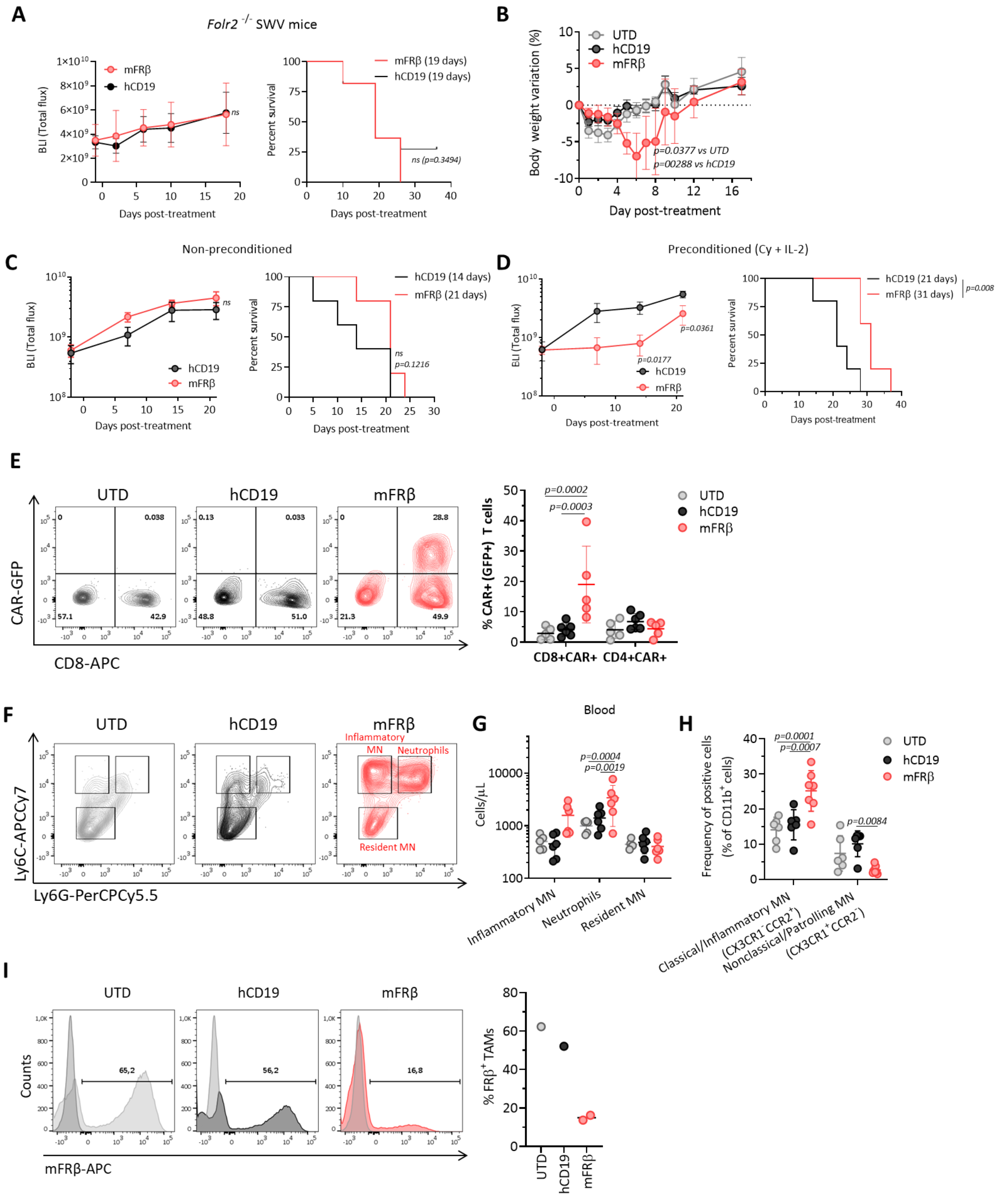
A
Folr2 -/- SWV mice
mFRβ
hCD19
BLI (Total flux)
Days post-treatment
Percent survival
mFRβ (19 days)
hCD19 (19 days)
ns (p=0.3494)
B
UTD
hCD19
mFRβ
Body weight variation (%)
p=0.0377 vs UTD
p=00288 vs hCD19
Day post-treatment
C
Non-preconditioned
hCD19 (14 days)
mFRβ (21 days)
ns
p=0.1216
D
Preconditioned (Cy + IL-2)
hCD19 (21 days)
mFRβ (31 days)
p=0.008
p=0.0177
p=0.0361
E
UTD
hCD19
mFRβ
CAR-GFP
CD8-APC
% CAR+ (GFP+) T cells
p=0.0002
p=0.0003
CD8+CAR+
CD4+CAR+
F
Inflammatory MN
Neutrophils
Resident MN
Ly6C-APCCy7
Ly6G-PerCPCy5.5
G
Blood
Cells/μL
p=0.0004
p=0.0019
H
Frequency of positive cells (% of CD11b+ cells)
p=0.0001
p=0.0007
p=0.0084
Classical/Inflammatory MN (CX3CR1- CCR2+)
Nonclassical/Patrolling MN (CX3CR1+ CCR2-)
I
Counts
mFRβ-APC
65,2
56,2
16,8
% FRβ+ TAMs

Supplementary Figure 7. Depletion of FRβ^{+} TAMs *in vivo* by mouse FRβ CAR-T cells reeducates the TME. (**A**) SWV *Folr2*$^{-/-}$ mice were inoculated i.p. with 5x10^{6} ID8 RFP-fLuc ovarian cancer cells. 21 days later, mice were treated with a single i.p. dose of 8x10^{6} mouse CAR^{+} T cells preceded by an i.p. injection of 150mg/kg of cyclophosphamide (Cy) 24 hours before T cell administration, and accompanied by i.p. injections of 15μg of IL-2 on three consecutive days. Tumor progression as monitored by bioluminescence imaging (BLI, left panel) as mean radiance ± SEM and Kaplan-Meier survival curves (right panel) (n=11 per group). (**B-H**) C57BL/6 mice were inoculated i.p. with 5x10^{6} ID8 RFP-fLuc ovarian cancer cells. 21 days later, mice were treated with a single i.p. dose of 8x10^{6} mouse CAR^{+} T cells preceded by an i.p. injection of 150mg/kg of cyclophosphamide (Cy) 24 hours before T cell administration, and accompanied by i.p. injections of 15μg of IL-2 on three consecutive days. Mice were euthanized for analysis at indicated time points or maintained for tumor progression monitoring. (**B**) Percentage of body weight variation in treated mice following T cell administration. (**C**) Tumor progression in mice treated with hCD19 or mFRβ CAR-T cells in the absence of Cy + IL-2 preconditioning. BLI as mean radiance ± SEM (left panel) and Kaplan-Meier survival curves (right panel) are shown (n=5 per group). (**D**) Tumor progression in mice treated with hCD19 or mFRβ CAR-T cells and preconditioned with Cy + IL-2. BLI as mean radiance ± SEM (left panel) and Kaplan-Meier survival curves (right panel) are shown (n=5 per group). (**E**) Presence of CAR^{+}-T cells in the ascites 6 days after treatment. Representative flow plots showing CAR^{+} cells as detected by GFP (Y-axis) and CD8 (X-axis) expression. Cells were previously gated on live/CD45^{+}/CD3^{+} (left panel). Mean frequencies of CAR^{+} T cells ± SD (n=5 mice per group) are shown (right panel). (**F**) Phenotype of myeloid cells in the ascites of treated mice 6 days after T cell administration, based on Ly6C (Y-axis) and Ly6G (X-axis) expression. Representative flow plots showing the gating strategy for myeloid cell subsets (gated on live, CD45^{+}CD11b^{+}) including resident monocytes (Ly6C^{-}Ly6G^{-}), inflammatory monocytes (Ly6C^{+}Ly6G^{-}) and neutrophils (Ly6C^{int}Ly6G^{+}). (**G**) Concentration of different myeloid cell populations (as described in Fig. S7C) in the blood of mice 10 days after T cell administration. Mean ± SD are represented (UTD n=5 mice, hCD19 and mFRβ n=6 mice). (**H**) Frequencies of myeloid cell subsets based on CCR2 and CX3CR1 expression (pregated on live, CD45^{+}CD11b^{+}) including classical/inflammatory monocytes (CX3CR1^{-}CCR2^{+}) and nonclassical/patrolling monocytes (CX3CR1^{+}CCR2^{-}) in ascites 6 days after T cell administration (UTD and hCD19 n=6 mice, mFRβ n=7 mice). (**I**) mFRβ expression in peritoneal TAMs collected 8 days after CAR T cell treatment from experiment in *Cd8* KO mice shown in Fig. 5N. Histograms (left panel) and frequencies of FRβ^{+} TAMs (pregated as live, CD45^{+}CD11b^{+}F4/80^{+}, right panel) are shown (UTD, hCD19 n=1 mouse, mFRβ n=2 mice). *p-values* by a (**B**, **E**, **G**, **H**) two-way ANOVA with Tukey's multiple comparison test, a (**A**, **C**, **D** (left panel)) two-tailed multiple t-test, or a (**A**, **C**, **D** (right panel)) two-sided Log-rank Mantel-Cox test are indicated. *ns*, non-significant; MN, monocytes. Source data are provided as a Source Data file.

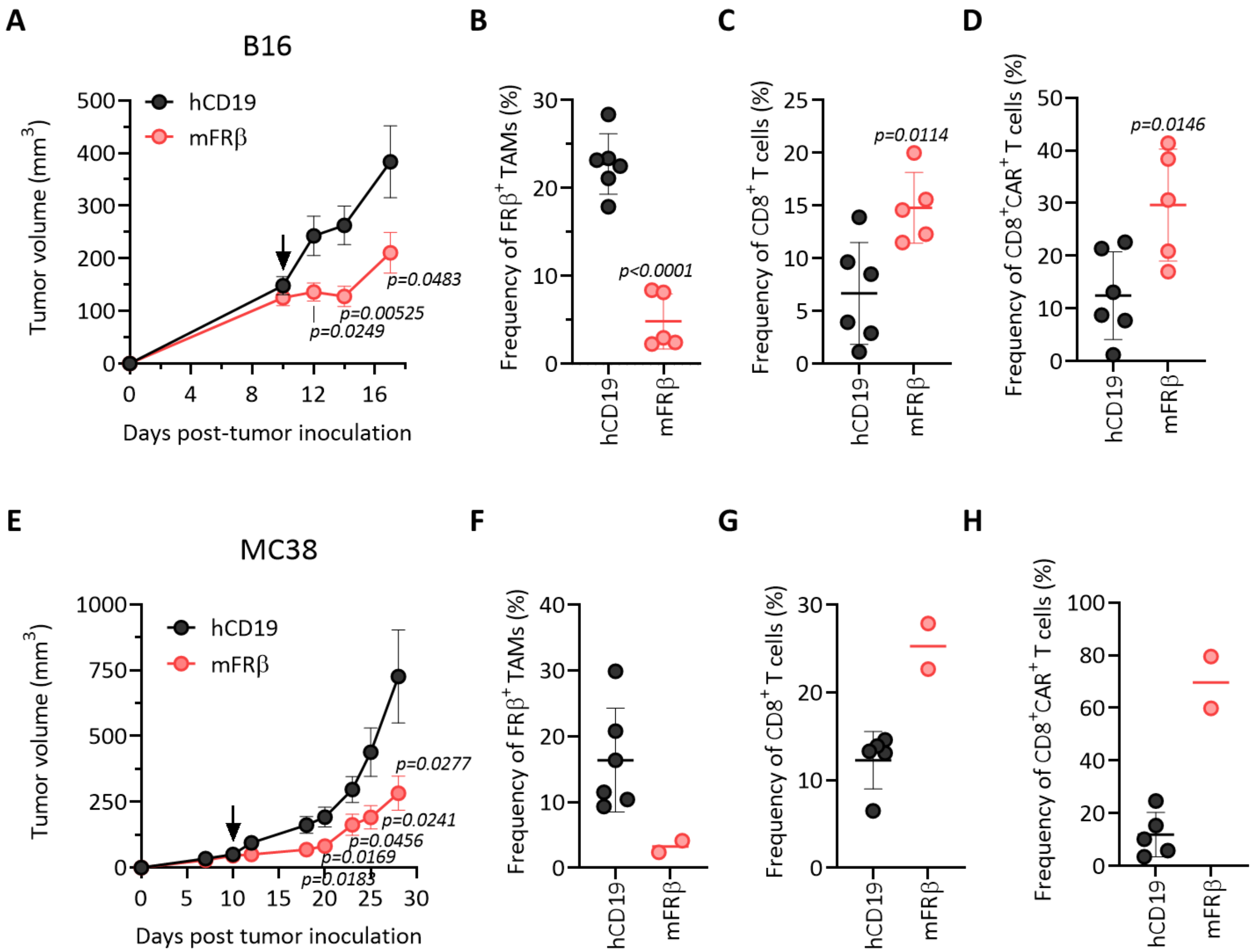

Supplementary Figure 8. Depletion of FRβ$^+$ TAMs by mouse FRβ CAR-T cells *in vivo* delays tumor growth and increases T cell infiltration in different solid tumor models. C57BL/6 mice were inoculated s.c. with 0.25x10^6 B16 melanoma or 0.5x10^6 MC38 colon adenocarcinoma cells. 10 days later, mice were randomized in groups and treated with a single i.v. dose of 8x10^6 mouse CAR$^+$-T cells preceded by an i.p. injection of 150mg/kg of cyclophosphamide (Cy) 24 hours before T cell administration, and accompanied by i.p. injections of 15µg of IL-2 on three consecutive days. Mice were monitored for tumor progression by caliper measurement. Arrows indicate time of treatment. Tumor volumes are represented for (**A**) B16 or (**E**) MC38 *in vivo* models. Data is represented as mean ± SEM (B16: hCD19 n=13, mFRβ n=11 tumors per group; MC38 n=12 tumors per group). Frequencies of intratumoral (**B**, **F**) FRβ$^+$ TAMs (defined as live, CD45$^+$CD11b$^+$F4/80$^+$ cells), (**C**, **G**) CD8$^+$ T cells (pregated on live, CD45$^+$) and (**D**, **H**) CD8$^+$CAR$^+$ T cells (defined as live, CD45$^+$CD8$^+$GFP$^+$) in mechanically dissociated tumors collected 7 days after CAR-T cell treatment are shown as mean ± SD for n>3. (**B**-**D**) hCD19 n=6, mFRβ n=5 tumors per group. (**F**-**H**) hCD19 n=5, mFRβ n=2 tumors per group. *p-values* by unpaired two-tailed multiple student's t-test are indicated. Source data are provided as a Source Data file.

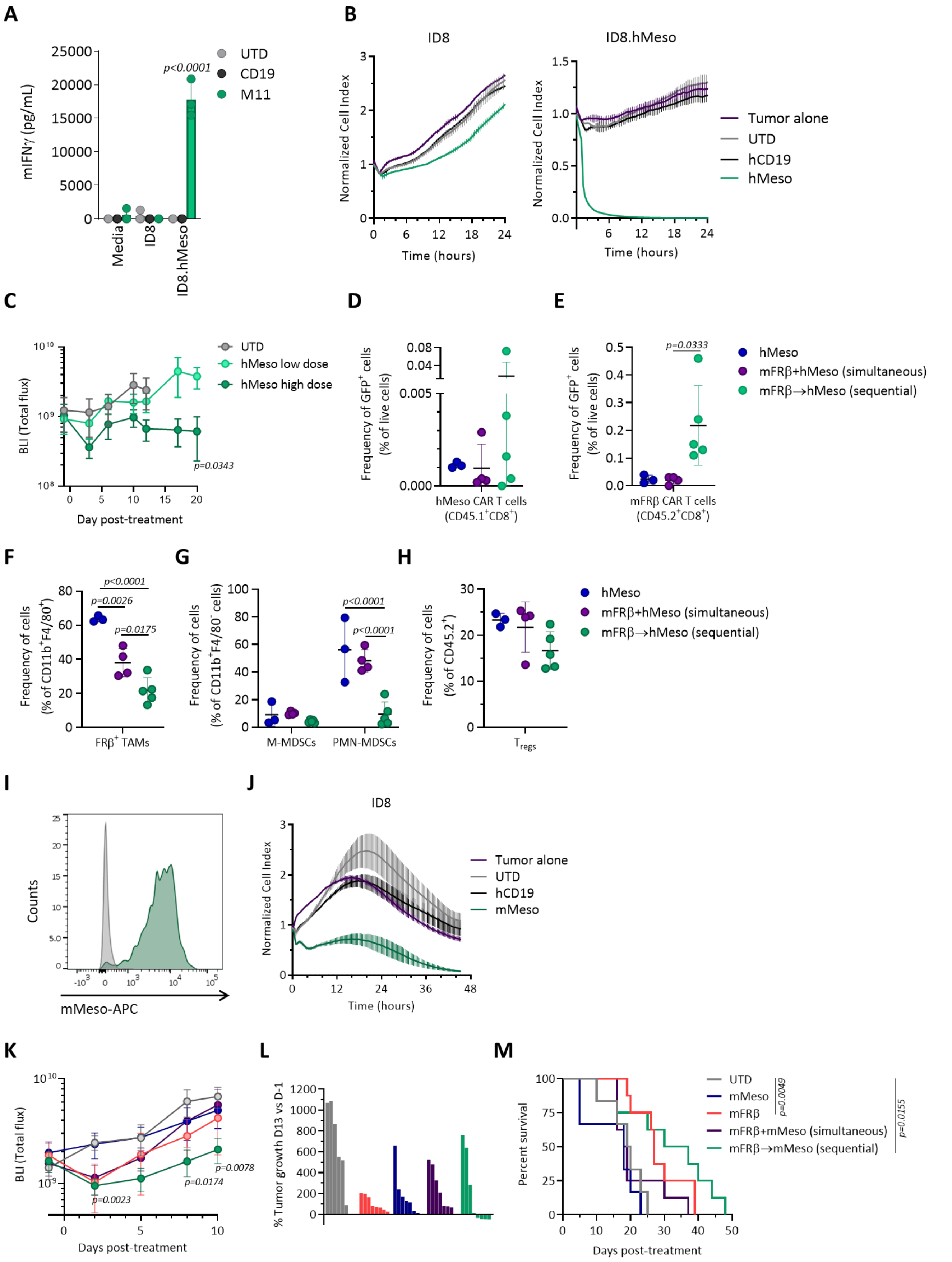
A
mIFNγ (pg/mL)
25000
20000
15000
10000
5000
0
p<0.0001
UTD
CD19
M11
Media
ID8
ID8.hMeso
B
ID8
ID8.hMeso
Normalized Cell Index
Time (hours)
Tumor alone
UTD
hCD19
hMeso
C
BLI (Total flux)
Day post-treatment
UTD
hMeso low dose
hMeso high dose
p=0.0343
D
Frequency of GFP+ cells (% of live cells)
hMeso CAR T cells (CD45.1+CD8+)
E
p=0.0333
mFRβ CAR T cells (CD45.2+CD8+)
hMeso
mFRβ+hMeso (simultaneous)
mFRβ→hMeso (sequential)
F
Frequency of cells (% of CD11b+F4/80+)
p<0.0001
p=0.0026
p=0.0175
FRβ+ TAMs
G
Frequency of cells (% of CD11b+F4/80- cells)
p<0.0001
p<0.0001
M-MDSCs
PMN-MDSCs
H
Frequency of cells (% of CD45.2+)
Tregs
hMeso
mFRβ+hMeso (simultaneous)
mFRβ→hMeso (sequential)
I
Counts
mMeso-APC
J
ID8
Normalized Cell Index
Time (hours)
Tumor alone
UTD
hCD19
mMeso
K
BLI (Total flux)
Days post-treatment
p=0.0023
p=0.0174
p=0.0078
L
% Tumor growth D13 vs D-1
M
Percent survival
Days post-treatment
UTD
mMeso
mFRβ
mFRβ+mMeso (simultaneous)
mFRβ→mMeso (sequential)
p=0.0049
p=0.0155

Supplementary Figure 9. Human mesothelin and mouse mesothelin specific CAR-T cells display antigen-specific reactivity *in vitro* and improved performance *in vivo* when given after preconditioning the TME with mouse FRβ CAR-T cells. (**A**) Levels of secreted mIFN-γ as detected by ELISA in the supernatants of hMeso CAR-T cells following overnight co-culture with parental or hMeso-enginereed ID8 target cells. Co-cultures were established at a 1:1 E:T ratio. Mean ± SD of triplicates is represented. (**B**) Real time cytotoxicity assays of hMeso CAR-T cells against ID8 (left panel) and ID8.hMeso (right panel) cells at 1:1 E:T ratio performed using xCelligence technology. Mean ± SD of triplicates is represented. (**C**) C57BL/6 mice were inoculated i.p. with $5x10^6$ ID8.hMeso-RFP-fLuc ovarian cancer cells. 21 days later, mice were treated with a single i.p. dose of $4x10^6$ (low dose) or $8x10^6$ (high dose) hMeso mouse CAR^+ T cells, or $8x10^6$ UTD T cells, accompanied by Cy+IL-2 preconditioning as indicated. Tumor progression as monitored by BLI is shown as mean radiance ± SEM (UTD n=3 mice, low dose n=4 mice, high dose n=5 mice). Frequency of (**D**) hMeso CAR^+ T cells ($CD45.1^+GFP^+$) or (**E**) mFRβ CAR^+ T cells ($CD45.2^+GFP^+$) in the peritoneal cavity of mice from the study depicted in **Fig. 6A** at the endpoint (day 56 post-administration for hMeso and mFRβ+hMeso groups and day 69 for mFRβ→hMeso). Frequencies of (**F**) $FRβ^+$ TAMs (**G**) MDSCs (M-MDSCs: $Ly6C^+Ly6G^-$, PMN-MDSCs: $Ly6C^{int}Ly6G^+$) and (**H**) Tregs ($CD4^+CD25^+$) in the peritoneal cavity of mice from the study depicted in **Fig. 6A** at the endpoint. (**D**-**H**) Means ± SD (hMeso n=3 mice, mFRβ+hMeso n=4 mice, mFRβ→hMeso n=5 mice) are represented. (**I**) Endogenous levels of mouse mesothelin expression in parental ID8 cells as detected by flow cytometry. Representative histogram (grey-isotype, green-mMeso) is shown. (**J**) Real time cytotoxicity assays of mMeso CAR-T cells against ID8 target cells at 10:1 E:T ratio performed using xCelligence technology. Mean ± SD of triplicates is represented. (**K-M**) C57BL/6 mice were inoculated i.p. with $5x10^6$ ID8 RFP-fLuc ovarian cancer cells. 21 days later, mice were treated with a single i.p. dose of $4x10^6$ hCD19, mFRβ or mMeso mouse CAR^+ T cells, or a combination of $4x10^6$ mFRβ and $4x10^6$ mMeso CAR^+ T cells given simultaneously or sequentially (on day 0 and day 3, respectively). Mice from groups treated with a single CAR received an equivalent dose of UTD T cells to even up the dose of treatment combination groups. T cell administration was preceded by an i.p. injection of 150mg/kg of cyclophosphamide (Cy) 24 hours earlier, and accompanied by i.p. injections of 15μg of IL-2 on three consecutive days. (**K**) Tumor progression as monitored by bioluminescence imaging is represented as mean radiance ± SEM is represented. (**L**) Waterfall plot showing the change in tumor bioluminiscence on day 13 versus baseline. Data from individual mice are represented. (**M**) Kaplan-Meier survival curves are represented. Endpoint was defined at animal body weight increase higher than 10% due to ascites formation. (**K**, **L**, **M**) Data represented for: UTD, hCD19, and hMeso n=6 mice; mFRβ, mFRβ+mMeso and mFRβ→mMeso n=8 mice. *p-values* by a (**A**, **D**-**H**) one-way ANOVA with Tukey's multiple comparison test, by a (**C**) two-tailed multiple t-test as compared to hMeso low dose, by a (**K**) two-tailed multiple t-test as compared to UTD or by a (**M**) two-sided Log-rank Mantel-Cox test are indicated. Source data are provided as a Source Data file.

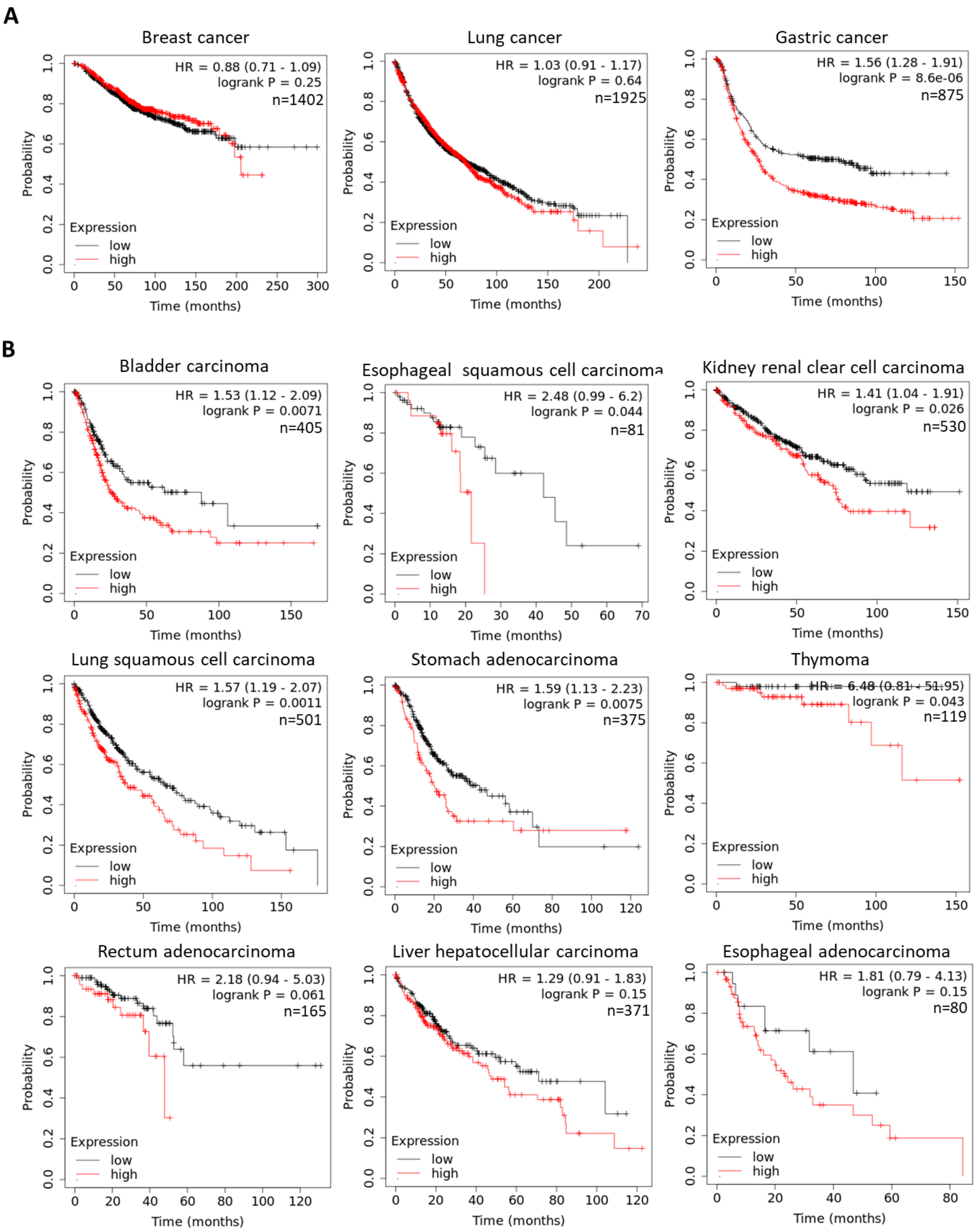

Supplementary Figure 10. Correlation of FRβ expression and survival in different human cancer types. (**A**) Overall survival curves for breast, lung and gastric cancer patients with high (red) or low (black) expression of FOLR2 (gene encoding for FRβ). The clinical outcome data

and the mRNA gene chip expression data was retrieved from the Kaplan-Meier plotter database. (**B**) Overall survival curves for patients with indicated cancer types with high (red) or low (black) expression of FOLR2 (gene encoding for FRβ). The clinical outcome data and the mRNA RNA seq gene expression data was retrieved from the Kaplan-Meier plotter database. N indicates number of patients included in the analysis for each stage. The Hazard Ratios (HR) and *p-values* by a two-sided Log-rank Mantel-Cox test are indicated.
