# Supplementary material for: CAR-T cell-mediated depletion of immunosuppressive tumor-associated macrophages promotes endogenous antitumor immunity and augments adoptive immunotherapy
Source: Nat Commun. 2021 Feb 9;12:877. doi: 10.1038/s41467-021-20893-2 (PMC7873057; doi:10.1038/s41467-021-20893-2)
Supplement: Supplementary file 3 — Descriptions of Additional Supplementary Files [file 41467_2021_20893_MOESM3_ESM.pdf]

Descriptions of Additional Supplementary Files

Supplementary Data 1

Description: List of differentially expressed genes in FRβ+ TAMs as compared to FRβ – TAMs as measured by RNAseq (n=4 per group). Adjusted p-value < 0.1 were considered.
