# Supplementary material for: CAR-T cell-mediated depletion of immunosuppressive tumor-associated macrophages promotes endogenous antitumor immunity and augments adoptive immunotherapy
Source: Nat Commun. 2021 Feb 9;12:877. doi: 10.1038/s41467-021-20893-2 (PMC7873057; doi:10.1038/s41467-021-20893-2)
Supplement: Supplementary file 5 — Reporting Summary [file 41467_2021_20893_MOESM5_ESM.pdf]

nature research

Corresponding author(s): Daniel J. Powell

Last updated by author(s): Dec 10, 2020

Reporting Summary

Nature Research wishes to improve the reproducibility of the work that we publish. This form provides structure for consistency and transparency in reporting. For further information on Nature Research policies, see our Editorial Policies and the Editorial Policy Checklist.

Statistics

For all statistical analyses, confirm that the following items are present in the figure legend, table legend, main text, or Methods section.

n/a	Confirmed	
☐	☒	The exact sample size (n) for each experimental group/condition, given as a discrete number and unit of measurement
☐	☒	A statement on whether measurements were taken from distinct samples or whether the same sample was measured repeatedly
☐	☒	The statistical test(s) used AND whether they are one- or two-sided<br>*Only common tests should be described solely by name; describe more complex techniques in the Methods section.*
☒	☐	A description of all covariates tested
☐	☒	A description of any assumptions or corrections, such as tests of normality and adjustment for multiple comparisons
☐	☒	A full description of the statistical parameters including central tendency (e.g. means) or other basic estimates (e.g. regression coefficient) AND variation (e.g. standard deviation) or associated estimates of uncertainty (e.g. confidence intervals)
☐	☒	For null hypothesis testing, the test statistic (e.g. F, t, r) with confidence intervals, effect sizes, degrees of freedom and P value noted<br>*Give P values as exact values whenever suitable.*
☒	☐	For Bayesian analysis, information on the choice of priors and Markov chain Monte Carlo settings
☒	☐	For hierarchical and complex designs, identification of the appropriate level for tests and full reporting of outcomes
☒	☐	Estimates of effect sizes (e.g. Cohen's d, Pearson's r), indicating how they were calculated

Our web collection on statistics for biologists contains articles on many of the points above.

Software and code

Policy information about availability of computer code

Data collection	Zeiss LSM 710, Illumina HiSeq 4000, Seahorse XFe96 Analyzer (Agilent), xCELLigence Real-Time Cell Analyzer System (ACEA Biosciences), IVIS Spectrum (Perkin Elmer), LSRFortessa (BD)
Data analysis	Illumina's bcl2fastq version v2.17.1.14 software, iDEP.90 RTCA Software 2.0 (ACEA Biosciences), LivingImage software v.4.3.1. (Caliper LifeSciences), GraphPad Prism 8.0 (GraphPad Software, Inc.), Excel 2016 (Microsoft), FlowJo v10.6.2 (FlowJo LLC), edgeR v3.2 software (Bioconductor)

For manuscripts utilizing custom algorithms or software that are central to the research but not yet described in published literature, software must be made available to editors and reviewers. We strongly encourage code deposition in a community repository (e.g. GitHub). See the Nature Research guidelines for submitting code & software for further information.

Data

Policy information about availability of data

All manuscripts must include a data availability statement. This statement should provide the following information, where applicable:

- Accession codes, unique identifiers, or web links for publicly available datasets
- A list of figures that have associated raw data
- A description of any restrictions on data availability

The authors declare that all data supporting the results in this study are available within the paper and its Supplementary Information. The RNAseq data have been deposited in the Gene Expression Omnibus (GEO) database, under accession number GSE155841 (https://www.ncbi.nlm.nih.gov/geo/query/acc.cgi?acc=GSE155841). For survival analysis of ovarian cancer patients, we used compiled datasets by Kaplan–Meier plotter (KM plotter). The following datasets were used in KM plotter: GSE14764, GSE15622, GSE18520, GSE19829, GSE23554, GSE26193, GSE26712, GSE27651, GSE30161, GSE3149, GSE51373, GSE63885, GSE65986, GSE9891, TGCA. Other data are available from the corresponding authors upon reasonable request. The source data underlying all main and

supplementary figures are provided as a Source Data file.

Field-specific reporting

Please select the one below that is the best fit for your research. If you are not sure, read the appropriate sections before making your selection.

☒ Life sciences ☐ Behavioural & social sciences ☐ Ecological, evolutionary & environmental sciences

For a reference copy of the document with all sections, see nature.com/documents/nr-reporting-summary-flat.pdf

Life sciences study design

All studies must disclose on these points even when the disclosure is negative.

Sample size	Although statistical methods were not used to predetermine sample size, sample sizes were chosen on the basis of estimates from pilot experiments. For in vitro studies, a minimum of triplicates was chosen to allow for calculation of statistics. For in vivo experiments, a sample size of 4-8 per treatment group was used which proved to be sufficient to reproducibly observe statistically significant differences.
Data exclusions	For in vivo studies, mice with extreme tumor burdens (either too high or too low compared to the average) were excluded from the experiment before CAR T cell transfer. Mice that received unsuccessful i.v. CAR T cell administrations were also excluded.
Replication	In vitro experiments were typically run with triplicate technical replicates and most of the experiments were reproduced at least twice. Most in vivo studies were performed twice with similar results. Some in vivo studies were performed once, although control treatment groups were repeated across many experiments and demonstrate reproducible effects. All attempts at replication were successful.
Randomization	For in vitro studies, samples were allocated to identical cell culture systems and there is no reason to believe that spatial organization influenced experimental outcomes. For in vivo studies, tumor burden was measured by bioluminiscence imaging (i.p. tumors) or by caliper (s.c. tumors) the same day or the day prior to CAR T cell administration. Mice were then randomized in treatment groups such that each group had the same overall average tumor burden (i.e. nostatistical differences among the groups via ANOVA).
Blinding	Tumor measurement by bioluminiscence imaging or by caliper was performed in a blinded fashion. Analysis of the data was performed in a non-blinded manner. Blinding was not necessary during data analysis since quantitative measures were used as much as possible (i.e. bioluminescence, tumor measurement, or body weight). In in vitro experiments, blinding was not necessary since all the samples were analyzed in the same way.

Reporting for specific materials, systems and methods

We require information from authors about some types of materials, experimental systems and methods used in many studies. Here, indicate whether each material, system or method listed is relevant to your study. If you are not sure if a list item applies to your research, read the appropriate section before selecting a response.

Materials & experimental systems

n/a	Involved in the study	
☐	☒	Antibodies
☐	☒	Eukaryotic cell lines
☒	☐	Palaeontology and archaeology
☐	☒	Animals and other organisms
☐	☒	Human research participants
☒	☐	Clinical data
☒	☐	Dual use research of concern

Methods

n/a	Involved in the study	
☒	☐	ChIP-seq
☐	☒	Flow cytometry
☒	☐	MRI-based neuroimaging

Antibodies

Antibodies used	Fc receptors were blocked by using TruStain FcX PLUS (anti-mouse CD16/32, #156603, 1:200 dilution) antibody as indicated by the manufacturer (Biolegend). The following antibodies were used in this study: human FRβ-APC (#391705, clone 94b, dilution 1:50), human CD206-PE (#321105, clone 15-2, dilution 1:50), human CD163-PerCPCy5.5 (#333607, clone GHI/61, dilution 1:50), mouse FRβ purified (#153302, clone 10/FR2, dilution 1:400), mouse FRβ-APC (#153305, clone 10/FR2, dilution 1:40), mouse CD45-FITC (#103107, clone 30-F11, dilution 1:200), mouse CD45-PE (#103105, clone 30-F11, dilution 1:80), mouse CD45-PacBlue (#103125, clone 30-F11, dilution 1:200), mouse CD45-APCCy7 (#103115, clone 30-F11, dilution 1:80), mouse CD45.1-Pacific Blue (#110722, clone A20, dilution 1:50), mouse CD45.2-BV711 (#109847, clone 104, dilution 1:80), mouse CD3-PECy7 (#100219, clone 17A2, dilution 1:80), mouse CD8α-APC (#100711, clone 53-6.7, dilution 1:80), mouse CD8α-APCCy7 (#100713, clone 53-6.7, dilution 1:20), mouse CD4-PE (#116005, clone RM4-4, dilution 1:80), mouse/human CD11b-PacBlue (#101223, clone M1/70, dilution 1:50), mouse/human CD11b-BV650 (#101237, clone M1/70, dilution 1:80), mouse F4/80-PECy7 (#123113, clone BM8, dilution 1:80), mouse CD206-AlexaF700 (#141733, clone C068C2, dilution 1:200), mouse PDL1-PercCPCy5.5 (#124333, clone 10F.9G2, dilution 1:20), mouse PD1-BV605 (#135219, clone 29F.1A12, dilution 1:160), mouse PDL2-PE (#107205, clone TY25, dilution 1:20), mouse Ly6C-APCCy7 (#128025, clone HK1.4, dilution 1:80), mouse Ly6G-PerCPCy5.5 (#127615, clone 1A8, dilution 1:80), mouse IFN-γ-PerCPCy5.5

(#505821, clone XMG1.2, dilution 1:20), mouse TNF-α-BV650 (#506333, clone MP6-XT22, dilution 1:40), goat anti-rat IgG-APC (#405407, clone Poly4054, dilution 1:80), mouse/rat/human FOXP3-AlexaF647 (#320014, clone 150D, dilution 1:20) (Biolegend), mouse CD204-PE (#130-120-811, clone REA148, dilution 1:50, Miltenyi), mouse Arg1-FITC (#IC5868F, dilution 1:10, R&D Systems), mouse Egr2-PE (#12-6691-82, clone erongr2, dilution 1:80, eBiosciences), iTAg Tetramer/PE-H2Kb OVA (SIINFEKL, #TB-5001-1, dilution 1:10, MBL), human mesothelin purified (#sc-33672, clone K1, dilution 1:100, SCBT), and mouse mesothelin purified (#LS-C179484-100, clone B35, dilution 1:100, LifeSpan Biosciences). The respective isotypes were also purchased. LIVE/DEAD Fixable Aqua dead cell stain kit (#L34957, dilution 1:1000, ThermoFisher Scientific) was used to assess viable cells.

Validation

Validation reports were provided by the antibody manufacturers (BioLegend, Miltenyi, R&D Systems, eBiosciences, MBL, SCBT or LifeSpan Biosciences).
Anti-human FRβ was validated in human peripheral blood monocytes: https://www.biolegend.com/en-us/products/apc-anti-human-folate-receptor-beta-fr-beta-antibody-15117
Anti-human CD206 was validated in GM-CSF-stimulated (day-3) human monocytes: https://www.biolegend.com/en-us/products/pe-anti-human-cd206-mmr-antibody-2994?GroupID=GROUP28
Anti-human CD163 was validated in Human peripheral blood lymphocytes, monocytes, granulocytes: https://www.biolegend.com/en-us/products/percp-cyanine5-5-anti-human-cd163-antibody-4794
Anti-mouse FRβ purified was validated in peritoneal macrophages collected from C57BL/6 mice 4 days after IP injection with thioglycolate: https://www.biolegend.com/en-us/products/purified-anti-mouse-folate-receptor-beta-fr-beta-antibody-14661
Anti-mouse FRβ-APC was validated in peritoneal macrophages from thioglycolate elicited BALB/c mouse: https://www.biolegend.com/en-us/products/apc-anti-mouse-folate-receptor-beta-fr-beta-antibody-15144
Anti-mouse CD45 was validated in C57BL/6 splenocytes: https://www.biolegend.com/en-us/products/fitc-anti-mouse-cd45-antibody-99
https://www.biolegend.com/en-us/products/pe-anti-mouse-cd45-antibody-100
https://www.biolegend.com/en-us/products/pacific-blue-anti-mouse-cd45-antibody-3102
https://www.biolegend.com/en-us/products/apc-cyanine7-anti-mouse-cd45-antibody-2530
Anti-mouse CD45.1 was validated in SJL mouse splenocytes: https://www.biolegend.com/en-us/products/pacific-blue-anti-mouse-cd45-1-antibody-3105
Anti-mouse CD45.2 was validated in C57/BL/6 splenocytes: https://www.biolegend.com/en-us/products/brilliant-violet-711-anti-mouse-cd45-2-antibody-13278
Anti-mouse CD3 was validated in C57/BL/6 splenocytes:
https://www.biolegend.com/en-us/products/pe-cyanine7-anti-mouse-cd3-antibody-6060
Anti-mouse CD8α was validated in C57/BL/6 splenocytes:
https://www.biolegend.com/en-us/products/apc-anti-mouse-cd8a-antibody-150
https://www.biolegend.com/en-us/products/apc-cyanine7-anti-mouse-cd8a-antibody-2269
Anti-mouse CD4 was validated in C57/BL/6 splenocytes:
https://www.biolegend.com/en-us/products/pe-anti-mouse-cd4-antibody-2499
Anti-mouse/human CD11b was validated in C57BL/6 mouse bone marrow cells:
https://www.biolegend.com/en-us/products/pacific-blue-anti-mouse-human-cd11b-antibody-3863
https://www.biolegend.com/en-us/products/brilliant-violet-605-anti-mouse-human-cd11b-antibody-7637
Anti-mouse F4/80 was validated in Thioglycolate-elicited BALB/c mouse peritoneal macrophages:
https://www.biolegend.com/en-us/products/pe-cyanine7-anti-mouse-f4-80-antibody-4070
Anti-mouse CD206 was validated in Thioglycollate-elicited BALB/c peritoneal macrophages:
https://www.biolegend.com/en-us/products/alexa-fluor-700-anti-mouse-cd206-mmr-antibody-13456
Anti- mouse PDL1 was validated in C57/BL/6 splenocytes: https://www.biolegend.com/en-us/products/percp-cyanine5-5-anti-mouse-cd274-b7-h1-pd-l1-antibody-13667
Anti- mouse PD1 was validated in Con-A stimulated C57BL/6 splenocytes (3 days): https://www.biolegend.com/en-us/products/brilliant-violet-605-anti-mouse-cd279-pd-1-antibody-7648
Anti-mouse PDL2 was validated in mB7-DC transfected cells:
https://www.biolegend.com/en-us/products/pe-anti-mouse-cd273-b7-dc-pd-l2-antibody-2547
Anti-mouse Ly6C was validated in C57BL/6 mouse bone marrow cells:
https://www.biolegend.com/en-us/products/apc-cyanine7-anti-mouse-ly-6c-antibody-6758
Anti-mouse Ly6G was validated in C57BL/6 mouse bone marrow cells:
https://www.biolegend.com/en-us/products/apc-cyanine7-anti-mouse-ly-6c-antibody-6758
Anti-mouse IFN-γ was validated PMA/Ionomycin-stimlated (6hrs) C57BL/6 splenocytes:
https://www.biolegend.com/en-us/products/percp-cyanine5-5-anti-mouse-ifn-gamma-antibody-4433
Anti- mouse TNF-α was validated PMA/Ionomycin-stimlated (6hrs) C57BL/6 splenocytes:
https://www.biolegend.com/en-us/products/brilliant-violet-650-anti-mouse-tnf-alpha-antibody-8829
Anti-mouse/rat/human FOXP3 was validated in C57/BL/6 splenocytes:
https://www.biolegend.com/en-us/products/alexa-fluor-647-anti-mouse-rat-human-foxp3-antibody-2892
Anti-mouse CD204 was validated in thioglycolate-elicited peritoneal exudate cells from C57BL/6 mice:
https://www.miltenyibiotec.com/US-en/products/cd204-antibody-anti-mouse-reafinity-rea148.html#pe:30-ug-in-200-ul
Anti-mouse Arg1 was validated in in HepG2 Human Cell Line and in Hepa 1-6 Mouse Cell Line:
https://www.rndsystems.com/products/human-mouse-arginase-1-arg1-fluorescein-conjugated-antibody_ic5868f
Anti-mouse Egr2 was validated in C57Bl/6 splenocytes stimulated overnight with plate-bound Anti-Mouse CD3e Functional Grade Purified:
https://www.thermofisher.com/antibody/product/EGR2-Antibody-clone-erongr2-Monoclonal/12-6691-82
Anti-human mesothelin was validated in ES-2 cells:
https://www.scbt.com/p/mesothelin-antibody-k1
Anti-mouse mesothelin was validated in: https://pubmed.ncbi.nlm.nih.gov/29618658/
Antibodies were validated in each experiment by using positive and negative cells or isotype controls. Appropriate compensation

controls were used for every experiment. The BD LSRFortessa was calibrated daily using CS&T beads (BD Biosciences).

Eukaryotic cell lines

Policy information about cell lines

Cell line source(s)	The retroviral packaging cell line PlatE with ecotropic envelope and the murine ovarian carcinoma cell lines ID8 and ID8.OVA were kindly provided by George Coukos from the University of Pennsylvania. PlatE were originally developed from 293T cells and commercially available. ID8 cell line was originally developed at the University of Kansas Medical Center, and donated to Dr. Coukos by Dr. K.F.Roby. ID8 RFP-fuc, ID8.mFRb RFP-fLuc, and ID8.hMeso RFP-fLuc cell lines were engineered in the lab. MC38 colon adenocarcinoma cell line was purchased from Kerafast. B16-F10 melanoma cell line was purchased from ATCC.
Authentication	For purchased cell lines COA was provided by the manufacturer. Commercial cell lines were not authenticated. For cell lines engineered in the lab, pertinent properties were confirmed by flow cytometry (e.g. mFRb, hMeso, mMeso, RFP).
Mycoplasma contamination	Cell lines were routinely tested for mycoplasma contamination (Universal Mycoplasma Kit Detection, ATCC). All the cell lines used in this study were negative for mycoplasma contamination.
Commonly misidentified lines (See ICLAC register)	None of the cell lines used in this study are listed in the ICLAC Database of Cross-contaminated or Misidentified Cell Lines.

Animals and other organisms

Policy information about studies involving animals; ARRIVE guidelines recommended for reporting animal research

Laboratory animals	C57BL/6 mice were purchased from Charles River. B6.129S2-Cd8tm1Mak (CD8 KO) and B6.SJL-Ptprca Pepcb/BoyJ (CD45.1) mice were purchased from The Jackson Laboratory. Females aged 6-8 weeks were used for the studies. SWV FRb KO or WT mice were kindly provided by Richard H. Finnel (Baylor College of Medicine, Houston) and breed in house. Males and females aged 6-8 weeks were used for the studies.
Wild animals	No wild animals were used in this study.
Field-collected samples	No field collected samples were used in this study.
Ethics oversight	All in vivo studies were conducted under IACUC approved protocols under established policies at the University of Pennsylvania.

Note that full information on the approval of the study protocol must also be provided in the manuscript.

Human research participants

Policy information about studies involving human research participants

Population characteristics	Randomly selected, de-identified ascites samples from ovarian cancer patients were purchased from the University of Pennsylvania Tumor BioTrust Collection.
Recruitment	University of Pennsylvania patients undergoing surgery, chemotherapy or other treatment for malignant gynecological tumors were elegible to donate biospecimens. No exclusions were made based on age, race or economic status of patients.
Ethics oversight	Ascites biospecimens were collected under an IRB approved research protocol (IRB702679).

Note that full information on the approval of the study protocol must also be provided in the manuscript.

Flow Cytometry

Plots

Confirm that:

- [x] The axis labels state the marker and fluorochrome used (e.g. CD4-FITC).
- [x] The axis scales are clearly visible. Include numbers along axes only for bottom left plot of group (a 'group' is an analysis of identical markers).
- [x] All plots are contour plots with outliers or pseudocolor plots.
- [x] A numerical value for number of cells or percentage (with statistics) is provided.

Methodology

Sample preparation	TAMs were obtained from the ascites of mice bearing i.p. ID8 tumors. At indicated time points after tumor inoculation, 10mL of PBS were injected i.p. and total cells in the wash were collected. Red blood cells were lysed using ACK lysis buffer (Thermo Fisher). For flow cytometry assays, 1-2x10^6 total cells were stained and analyzed as described below. For in vitro co-culture assays, cells were labeled with CD11b MicroBeads (mouse/human) (Miltenyi Biotec) and isolated with LS MACS separation

	columns according to the manufacturer's instructions. For some experiments, CD11b+ cells were stained for markers CD45 and F4/80, and singlets, live, double positive cells, were sorted based upon FR expression by the Flow Cytometry and Cell Sorting Facility (UPenn). Spleens were collected and splenocytes were dissociated and pushed through a 70µm cell strainer. Red blood cells were lysed using ACK lysis buffer. Solid tumors were collected at indicated time points and mechanically dissociated using GentleMACs tubes and dissociator according to manufacturer's recommendations (Miltenyi Biotec). Single cell suspensions were stained by using standard flow cytometry protocols. Cells were washed before and after staining, and Fc block (TruStain FcX PLUS) was used. Blood was obtained and stained by using Trucount tubes (BD Biosciences).
Instrument	Flow cytometry was performed on a Fortessa-LSR II cytometer (Becton-Dickinson). Sorting was performed on a FACS Aria II SORP (Becton-Dickinson).
Software	FCS files were analyzed with FlowJo v10.6.2.
Cell population abundance	Target cell lines were sorted for >99% RFP/target antigen positivity, and TAMs were sorted for >99% FRb positivity/negativity on a BD Aria sorter by staff of the Flow Cytometry and Cell Sorting Facility (UPenn). Purity was determined by flow cytometry during and after the sort.
Gating strategy	FACS gating was performed as follows: FSC/SSCsingletslive cells (L/D Aqua negative)gating of interest. Boundaries between positive and negative were established by using appropriate controls (i.e. isotypes). For gating on CAR expression, untransduced T cells were used to draw the gates of interest.

☒ Tick this box to confirm that a figure exemplifying the gating strategy is provided in the Supplementary Information.
